# Using Steered Molecular Dynamics to Predict and Assess Hsp70 Substrate-Binding Domain Mutants that Alter Prion Propagation

**DOI:** 10.1371/journal.pcbi.1002896

**Published:** 2013-01-31

**Authors:** Linan Xu, Naushaba Hasin, Manli Shen, Jianwei He, Youlin Xue, Xiaohong Zhou, Sarah Perrett, Youtao Song, Gary W. Jones

**Affiliations:** 1 Province Key Laboratory of Animal Resource and Epidemic Disease Prevention, Liaoning University, Shenyang, China; 2 Department of Biology, National University of Ireland Maynooth, Maynooth, County Kildare, Ireland; 3 School of Environmental Science, Liaoning University, Shenyang, China; 4 National Laboratory of Biomacromolecules, Institute of Biophysics, Chinese Academy of Sciences, Beijing, China; Scuola Internazionale Superiore di Studi Avanzati, Italy

## Abstract

Genetic screens using *Saccharomyces cerevisiae* have identified an array of cytosolic Hsp70 mutants that are impaired in the ability to propagate the yeast [*PSI* ^+^] prion. The best characterized of these mutants is the Ssa1 L483W mutant (so-called *SSA1-21*), which is located in the substrate-binding domain of the protein. However, biochemical analysis of some of these Hsp70 mutants has so far failed to provide major insight into the specific functional changes in Hsp70 that cause prion impairment. In order to gain a better understanding of the mechanism of Hsp70 impairment of prions we have taken an *in silico* approach and focused on the *Escherichia coli* Hsp70 ortholog DnaK. Using steered molecular dynamics simulations (SMD) we demonstrate that DnaK variant L484W (analogous to *SSA1-21*) is predicted to bind substrate more avidly than wild-type DnaK due to an increase in numbers of hydrogen bonds and hydrophobic interactions between chaperone and peptide. Additionally the presence of the larger tryptophan side chain is predicted to cause a conformational change in the peptide-binding domain that physically impairs substrate dissociation. The DnaK L484W variant in combination with some *SSA1-21* phenotypic second-site suppressor mutations exhibits chaperone-substrate interactions that are similar to wild-type protein and this provides a rationale for the phenotypic suppression that is observed. Our computational analysis fits well with previous yeast genetics studies regarding the functionality of the Ssa1-21 protein and provides further evidence suggesting that manipulation of the Hsp70 ATPase cycle to favor the ADP/substrate-bound form impairs prion propagation. Furthermore, we demonstrate how SMD can be used as a computational tool for predicting Hsp70 peptide-binding domain mutants that impair prion propagation.

## Author Summary

Direct non-covalent interactions between protein substrates and molecular chaperones play crucial roles in the protein folding process. [*PSI^+^*] is a prion of the yeast *Saccharomyces cerevisiae*, which is formed by mis-folding of the native Sup35 protein in a process analogous to formation of prions in mammals. While much genetic data exists showing a clear role for Hsp70 in prion propagation, biochemical data has yet to provide a clear link to how Hsp70 functions in prion propagation or how some Hsp70 mutants successfully impair *in vivo* propagation of prions. This paper employs a novel simulation method termed “steered molecular dynamics” to explore the different types and amounts of non-covalent interactions between wild type and mutated Hsp70, with a model substrate. Extrapolating the *in silico* data allowed us to decipher how a mutant Hsp70 impairs yeast prion propagation and allows us to predict other Hsp70 mutants that should behave in the same manner and to test these predictions in a yeast-based system. Our computational data shows that increasing the binding affinity of Hsp70 for substrate is one way of impairing prion propagation, a proposal that correlates very well with previous experimental genetic data.

## Introduction

Hsp70 is a highly conserved essential molecular chaperone that assists protein folding in general and protects cells from stress by preventing aggregation of stress-denatured proteins [1]. Hsp70 proteins consist of two functional domains: the N-terminal region comprises the nucleotide binding domain, and the C-terminal region the substrate-binding domain, which consists of a β-sheet rich substrate-binding cavity and a α-helical region that forms a lid over this cavity [2], [3]. N-terminal ATP hydrolysis induces a conformational change in the protein that closes the lid, trapping the substrate [3]. Additionally, binding of substrate causes a conformational change within the substrate-binding domain that stimulates ATP hydrolysis in proportion to its binding affinity, thus demonstrating a clear inter-domain communication mechanism for regulating Hsp70 function [4], [5].

In line with yeast prions being viewed as a protein-folding problem, altered abundance or function of a variety of molecular chaperones can affect their propagation [6]. Hsp104, a molecular chaperone that protects cells from exposure to stress by resolubilizing denatured protein from aggregates, is thought to facilitate yeast prion [*PSI^+^*] “seed” generation by breaking Sup35p aggregates into more numerous pieces [7]–[9]. Protein disaggregation by Hsp104 requires the assistance of Hsp70, which normally acts in [*PSI^+^*] “seed” propagation by dismantling polymers from Sup35 aggregates [10]. *S. cerevisiae* has four cytosolic Hsp70s of the Ssa subfamily, and expression of at least one of them is essential for growth [11]. Ssa1 is one of two constitutively expressed Ssa Hsp70s. Previous studies have identified and characterized a dominant mutant of Ssa1^L483W^ (Ssa1-21) that impairs propagation of the yeast [*PSI^+^*] prion, weakening the phenotype caused by [*PSI^+^*] and causing a reduction in the number of [*PSI^+^*] “seeds” per cell [12], [13]. Despite the considerable impairment of [*PSI^+^*], Ssa1-21 has little effect on cell growth under optimal or stressful conditions, which therefore provides a convenient model to study yeast prion regulation *in vivo* [14], [15]. Previous *in vivo* studies suggested that Ssa1-21 has enhanced substrate binding compared with wild-type Ssa1 [14]. Due to the location of residue L483 in the substrate-binding domain, Ssa1-21 might bind more avidly to Sup35 aggregates and thus impair the ability Hsp70 to promote the disassembly of aggregates into smaller oligomers [14]. This hypothesis was further supported by observations using fluorescence recovery after photobleaching (FRAP) and semi-denaturing detergent-agarose gel electrophoresis (SDD-AGE) demonstrating that Sup35 aggregates are larger in *SSA1-21* [*PSI^+^*] compared to wild type cells [13]. A recent *in vitro* study suggests that Ssa1-21 actually has weakened substrate-binding activity towards a small peptide substrate compared with wild-type Ssa1 [16]. The apparent discrepancy between the *in vivo* and *in vitro* activities of the Ssa1-21 protein may simply reflect the limitations of the biochemical assay used to mimic interaction with a prion substrate *in vivo*.

Molecular dynamics (MD) simulation is a classical method, which can provide detailed information on structural changes. However, applying such a technique to studying SBD- substrate interactions for Hsp70 would provide minimal information as the complete release of substrate from the SBD hydrophobic pocket would not be accomplished in the time scale of a conventional MD simulation. Steered molecular dynamics (SMD) is a way to imitate atomic force microscopy (AFM) experiments and can provide insight to the molecular mechanisms underlying the dissociation process of a protein and associated substrate [17], [18]. SMD simulations have been successfully used to investigate ligand-receptor interactions and the unfolding process of proteins [17]–[20]. We therefore applied the powerful SMD simulation technique to study the dissociation process of substrate from the SBD hydrophobic binding pocket of the highly conserved bacterial Hsp70 protein DnaK. DnaK is the most intensively studied Hsp70 protein with a high quality crystal structure available as well as detailed biochemical data [3]–[5], [21]. Corresponding structural information is severely lacking for Ssa1. Additionally, justified correlations have been made between DnaK biochemical activities (wild-type and mutant variants) and mutants obtained in the yeast ortholog Ssa1 [4], [22], [23]. Here we demonstrate an excellent correlation between previous experimental data for DnaK- substrate binding and that obtained through SMD simulations. We also use SMD simulations to characterize a DnaK^L484W^ mutant that provides strong evidence suggesting that increased substrate binding is the reason behind the prion-impairing ability of the equivalent yeast Ssa1 mutant. Additionally we demonstrate that SMD can be used to identify and predict Hsp70 SBD mutants that will impair prion propagation *in vivo*. We further validate our approach of analyzing the well-characterized DnaK system by demonstrating that SMD simulations carried out on computer-modeled wild type and mutant Ssa1 proteins behave in a strikingly similar manner to their DnaK counterparts.

## Results

### MD equilibrium simulations for DnaK^WT^ and DnaK^L484W^

The system temperature was coupled at 300K and the total energy fluctuated within less than 0.7% following short-time equilibration. The structural stability of models was assessed by calculation of the root-mean-square deviation (RMSD). To select equilibrated states as input structures for the SMD simulations, the overall RMSDs of Cα atoms of the protein were calculated as a function of the simulation time (Figure S1). The RMSD profiles indicate that the conformation of the proteins was equilibrated at approximately 2.5 ns and the values of RMSD for both DnaK^WT^ and DnaK^L484W^ were generally stabilized near 0.25 nm. The stable structures of each of the proteins provide appropriate starting points for subsequent SMD simulations and is also suggestive of similar structural stabilities for both proteins.

### Determination of the pulling velocity and spring constant

In constant velocity SMD (cv-SMD), the value of the spring force varies significantly depending on what pulling velocity and spring constant were applied to the system [24]–[26]. Higher pulling velocities result in dis-equilibrium, which introduces significant error into the simulation results [25]. To mimic AFM experiments as much as possible, the pulling velocity should be as small as possible [27]. We carried out 12 different pulling velocity simulations (ranging from 0.001 nm/ps to 0.08 nm/ps) to identify a suitable velocity in this DnaK system. Figure S2A summarizes the results of the rupture forces caused by different pulling velocities. The rupture force was defined as the highest peak value of the pulling force. There was an apparent linear dependency between rupture force and pulling velocity when velocities were lower than 0.03 nm/ps. For subsequent SMD simulations a pulling velocity of 0.001 nm/ps was chosen, as the external harmonic potential has no obvious influence on the structure of the protein.

Another important consideration for SMD simulations is that the spring constant must be large enough to enable the local dissociation potential to be measured; however it must not be too large as this leads to difficulty in measurements due to high-levels of background noise [27]. In this study, we assessed six different spring constants (2000, 1000, 500, 300, 100, 50 kJ/mol/nm^2^). Figure S2B shows that under the same pulling velocity, the spring constants of 2000, 1000 and 500 kJ/mol/nm**^2^** caused large fluctuations in rupture force, while spring constants of 100 and 50 kJ/mol/nm**^2^** had minimal effect. A spring constant of 300 kJ/mol/nm^2^, however, produced a measurable and consistent level of rupture forces and was therefore chosen for subsequent SMD simulations.

### Interaction energy analysis of the substrate dissociation process

**Figure 1:**
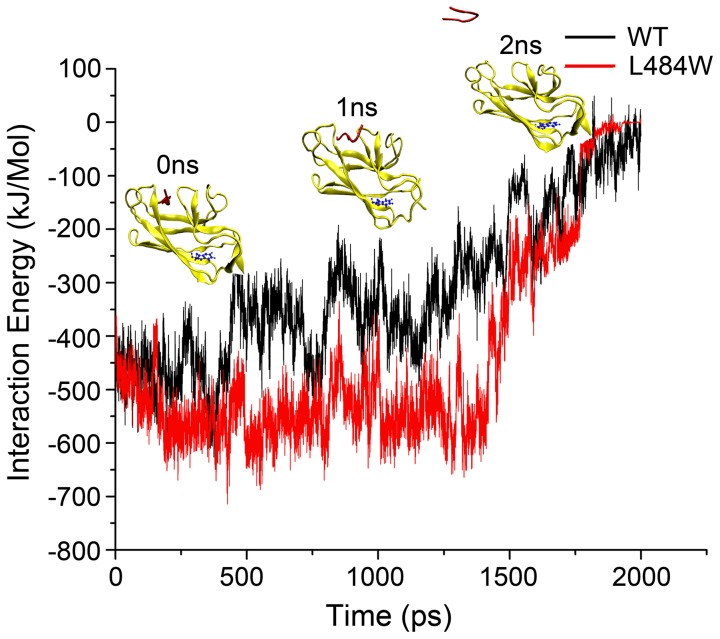
The substrate release process of DnaK during 2 ns SMD. Ribbon schematic representations of the DnaK SBD (393–507) with the peptide NRLLLTG, used in the SMD simulations. The simulated pulling of substrate from the binding cleft of DnaK was carried out over a 2000 ps time period. The DnaK SBD is yellow, substrate is red, and the residue 484 tryptophan is blue. The graph records changes in interaction energy during the course of the SMD simulation with DnaK^L484W^ (red) and DnaK^WT^ (black).

The release of substrate from the cleft of a protein or enzyme is a dynamic process, which can be analyzed by measuring the interaction energy [27]. Following equilibration of the DnaK substrate bound systems and applying the appropriate previously derived parameters, we analyzed the interaction energy profiles for DnaK^WT^ and DnaK^L484W^ with substrate, during the simulated process of pulling substrate from the SBD cleft. Figure 1 shows a diagrammatic representation of the process and also shows the fluctuations in interaction energy for DnaK^WT^ and DnaK^L484W^ during the 2 ns SMD simulations. With the application of steering forces to the system dis-equilibrium is achieved by the first time-point. Following the time-course of the simulation, the interaction energy initially remained at a low level of about 1300 ps, which indicates the presence of strong interactions between peptide substrate and the DnaK SBD cleft during this dynamic procedure. However, as the substrate is eventually pulled away from the cleft, the energy curve quickly approaches zero. The energy curves for peptide dissociation can be roughly divided into two distinct phases: Phase I, when the peptide is located within the SBD binding site, which ranges from 0–1300 ps for DnaK^WT^ and 0–1400 ps for DnaK^L484W^; and Phase II, when the peptide is released into the bulk solvent, which ranges from 1300–2000 ps for DnaK^WT^ and 1400–2000 ps for DnaK^L484W^. The DnaK^L484W^-substrate complex clearly has a lower energy curve than the DnaK^WT^ complex, especially in Phase I, which indicates a more stable DnaK^L484W^ complex compared to DnaK^WT^. It therefore appears that although L484 is not located directly in the SBD cleft, this residue plays a key role in influencing DnaK substrate binding affinity.

### Hydrogen bond and hydrophobic interaction analyses during substrate release

**Figure 2:**
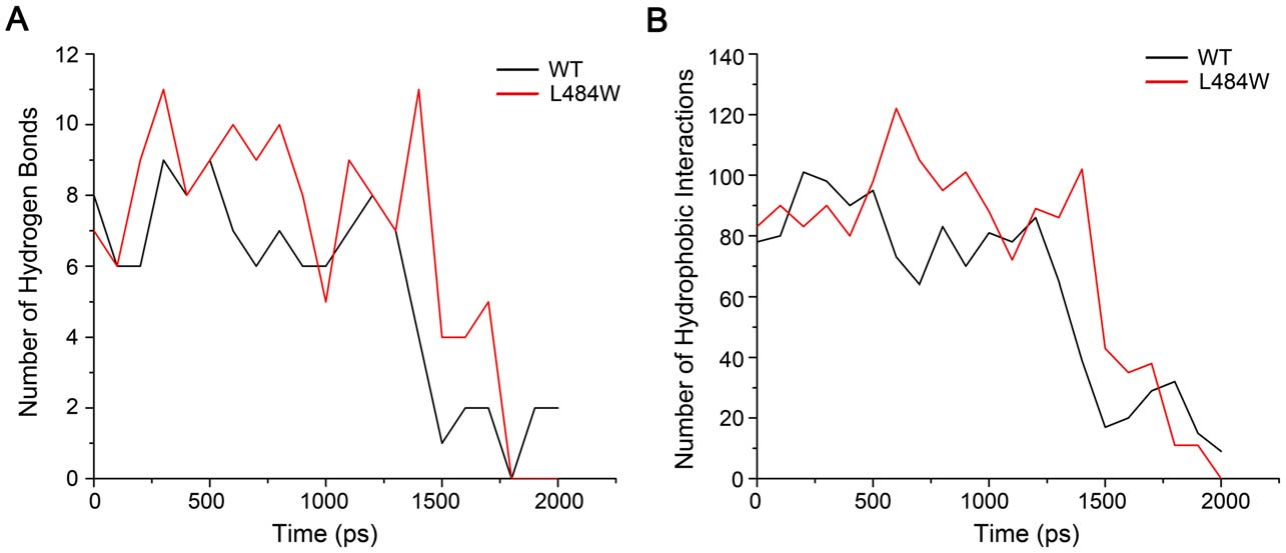
Changes in interaction forces as a function of simulation time. (A) Temporal evolution of the number of hydrogen bonds number formed between the SBD of DnaK and peptide under the application of force for DnaK^L484W^ (red) and DnaK^WT^ (black) complex; (B) Variations in the numbers of direct hydrophobic interactions of DnaK^L484W^ (red) and DnaK^WT^ (black) complexes in the SMD simulation, respectively. The calculation of interactions is recorded every 100 ps.

Hydrogen bonds and hydrophobic interactions (or van der Waals) are important non-covalent interactions in stabilizing protein structures. Previous experimental research has indicated that the peptide interacts with the SBD of DnaK through van der Waals forces and seven intermolecular hydrogen bonds [3]; these interactions are summarized in Table 1. In our simulation studies, these two types of non-covalent interactions were the only ones detected in the system. Figure 2A shows the total number of hydrogen bonds formed between the peptide and DnaK as a function of time during the simulation. According to this curve, we can clearly determine that during the simulation the total number of intermolecular hydrogen bond between DnaK^L484W^ and substrate is greater than that of DnaK^WT^. Moreover, in our SMDs five out of the seven major hydrogen bonds identified by Zhu *et al*. [3] contribute significantly to stabilizing the DnaK^WT^ complex while four contribute to DnaK^L484W^ complex stabilization (Table 1). However, while having a reduced number of hydrogen bond interactions compared to wild type, those formed between DnaK^L484W^ and substrate are in existence for a much longer time period during the time course of the simulation (Table 1). Three of these, Ala^429^(N)-Leu^904^(O), Ser^427^(N)-Arg^902^(O) and Ser^427^(O)-Leu^904^(N), are the most significant intermolecular hydrogen bonds influencing the dissociation processes. In addition, it was found that the lifetimes of one extra hydrogen bond (Thr^428^(OG1)-Thr^906^(OG1)) in the DnaK^WT^ complex, and three extra hydrogen bonds (Thr^409^(OG1)-Asn^901^(N), Thr^428^(OG1)-Thr^906^(OG1), Ala^435^(O)-Gly^907^(N)) in the DnaK^L484W^ complex, are longer by 50% in our simulations. This suggests these bonds may also play important roles in influencing the dissociation process (Table 1).

**Table 1:** Comparison of peptide-substrate interactions identified in SMD simulations with previous experimental data.

Peptide residue	Hydrogen bonds			Residues in van der Waals contacts		
	SMDs (WT)^a^	SMDs (L484W)^a^	Experimental^b^	SMDs (WT)^a^	SMDs (L484W)^a^	Experimental^b^
Asn^901^	-	*409OG1:901N* (60%)	-	-	-	-
Arg^902^	**427N:902O** (70%)	**427N:902O** (70%)	427N:902O	**Thr^403^, Phe^426^, Thr^409^**	**Thr^403^, Phe^426^, Thr^409^**	Thr^403^, Phe^426^, Thr^409^, Val^407^
Leu^903^	**404N:903O** (20%)	**404N:903O** (35%)	404N:903O	**Met^404^, Ala^429^, Ser^427^**	**Met^404^, Ala^429^, Ser^427^**	Met^404^, Ala^429^, Ser^427^
Leu^904^	**429N:904O** (75%), **427O:904N** (65%)	**429N:904O** (85%), **427O:904N** (85%)	429N:904O, 433NE2:904O, 427O:904N	**Phe^426^, Val^436^, Thr^403^**, *Thr^428^*	**Phe^426^, Val^436^, Ile^401^, Thr^403^, Ile^438^**, *Thr^428^*	Phe^426^, Val^436^, Ile^401^, Thr^403^, Ile^438^
Leu^905^	-	-	437N:905O	**Met^404^, Ala^429^**	**Met^404^, Ala^429^**	Met^404^, Ala^429^
Thr^906^	*428OG1:906OG1* (60%)	*428OG1:906OG1* (75%)	-	**Gln^433^**, *Thr^428^*	**Gln^433^**, *Thr^428^*	Gln^433^, Ala^435^
Gly^907^	**437OG:907N** (10%)	*435O:907N* (60%)	437OG:907N	-	-	-

a The main interactions that were detected during the SMD simulations are indicated by upright letters in bold. Other important interactions were the lifetime was greater than 50% are shown in italics. The lifetime of hydrogen bonds is described in parentheses.

b The main interactions identified experimentally by Zhu *et al* [3].

In this study the number of van der Waals contacts of nonpolar atoms represents the hydrophobic interaction strength [28]. As shown in Figure 2B, the average number of hydrophobic interactions of the DnaK^L484W^ complex is obviously greater than that of DnaK^WT^. Moreover, the rupture behavior of the hydrophobic interactions at the binding site in DnaK^L484W^ complex occurred later than that in the DnaK^WT^ complex, which is similar for the rupture behavior of the hydrogen bond network (Figures 2A and 2B). Moreover, in our SMDs most of hydrophobic interactions identified by Zhu *et al*. [3] contribute significantly to stabilizing the DnaK^WT^ and DnaK^L484W^ complexes (Table 1).

**Figure 3:**
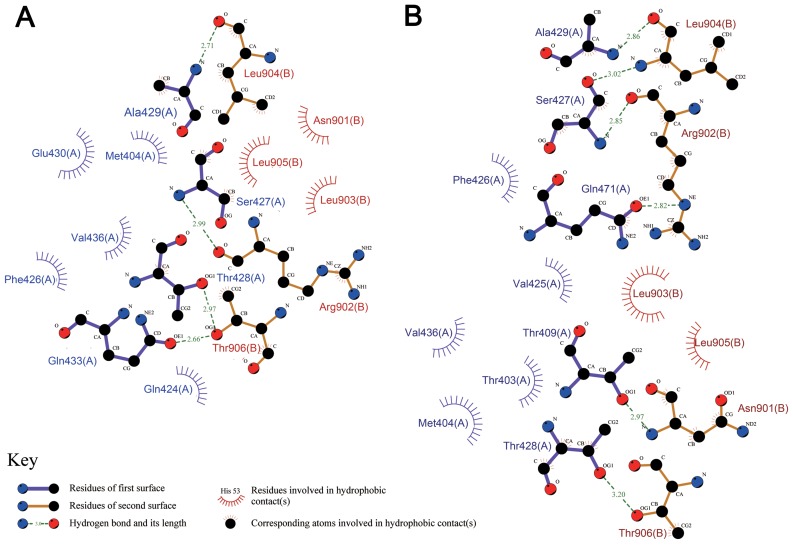
Interactions at the largest rupture force. Schematic diagrams showing hydrogen bonds and hydrophobic interactions between substrate and substrate-binding domain complexes for DnaK^WT^ (A) and DnaK^L484W^ (B) at the largest rupture force. The hydrophobic interactions (with distances of less than 3.5 Å) are shown as spiked spheres and dashed lines indicate hydrogen bonds.

To analyze in more detail the interactions between the substrate and the hydrophobic pocket, specific structural snapshots were taken at the largest rupture force point (DnaK^WT^ at 1300 ps, DnaK^L484W^ at 1400 ps). Hydrogen bonds and hydrophobic interactions between the substrate and binding pocket were analyzed using the LIGPLOT program. As shown in Figure 3 the three most important intermolecular hydrogen bonds, Ala^429^(N)- Leu^904^(O), Ser^427^(N)-Arg^902^(O) and Ser^427^(O)-Leu^904^(N) , were still formed in DnaK^L484W^ at this moment, whereas only two of them, Ala^429^(N)-Leu^904^(O) and Ser^427^(N)-Arg^902^(O), were formed in DnaK^WT^. Additionally, at this point there are six hydrogen bonds in the DnaK^L484W^ complex and only four in the case of DnaK^WT^. Furthermore, 101 hydrophobic interactions were detected between residues of the SBD (Thr403, Met404, Val425, Phe426, Ser427, Thr428, Ala429, Val436, Gln471) and substrate in DnaK^L484W^, especially Thr403, Met404, and Ser427, compared to only 81 hydrophobic interactions in DnaK^WT^ (formed between Met404, Gln424, Phe426, Ser427, Thr428, Ala429, Glu430, Val436 and substrate).

The detailed interaction analysis from this *in silico* work, as well as interaction analysis from previous experimental data is summarized in Table 1. The most significant interactions between SBD residues and substrate identified in this study are highly consistent with previous experimental data [3]. This fact validates the accuracy of the simulation and robustness of the data obtained.

### Conformational changes in the SBD in the L484W mutant

**Figure 4:**
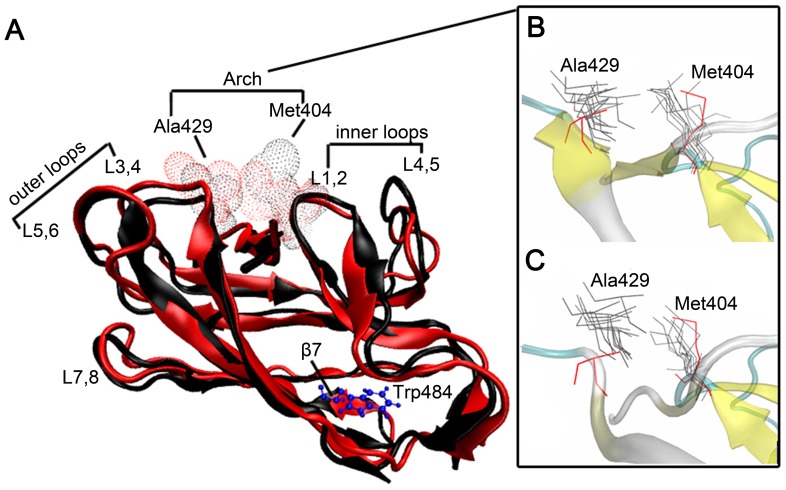
Superimposition of DnaK^WT^ and DnaK^L483W^ structures. (A) Superimposition of the Cα atoms of DnaK^L484W^ (red) and DnaK^WT^ (black) structures at the end of the MD simulation. (B) and (C) show superposition of the arch structure snapshots for DnaK^WT^ and DnaK^L484W^, respectively.

L484W is a mutation within the C-terminal region of DnaK. Previous work suggests that the equivalent mutation in yeast cytosolic Hsp70 causes increased substrate binding [14], a proposal that is clearly reflected in our binding analysis. Although L484W substitution is within the SBD, it is distant from the substrate-binding pocket. So its effect on the substrate-binding affinity may be indirect perhaps through conformational changes induced within the SBD. Previous studies have shown that the arch structure formed by residues Met404 and Ala429 of the two substrate contacting loops in the SBD of DnaK contributes significantly to peptide binding [3], [5], [21]. These two residues were also highlighted in both DnaK^WT^ and DnaK^L484W^ in our SMD simulations (Table 1, Figure 3). Using the VMD program we assessed potential structural changes within the SBD between DnaK^WT^ and DnaK^L484W^. The final structure (following MD simulations) of DnaK^L484W^ was superimposed with the corresponding final structure of DnaK^WT^ for the Cα (Figure 4A). We found that DnaK^L484W^ can more readily extend Ala429 to Met404 and then form a tighter arch than DnaK^WT^ (Figures 4B and 4C). It seems therefore that L484W, a tryptophan mutant in β7, somehow induces long-range conformational changes that result in the formation of a tighter arch structure that may impede efficient substrate release (Figure 4A).

### Analysis of other Leucine 484 mutations

In order to explore further the possible regulation mechanism of the L484W variant, we performed more MD and SMD simulations using different amino acid substitutions at L484, namely DnaK^L484A^ and DnaK^L484H^. Alanine has a similar hydrophobicity as tryptophan but has a much smaller side chain while histidine has a similar size of side chain as tryptophan but a much lower hydrophobicity. With these two “control” mutants, we can investigate whether the hydrophobicity and/or side chain structure may play important roles in the L484W variant. Compared with DnaK^WT^, DnaK^L484W^ and DnaK^L484H^, the DnaK^L484A^ mutant shows significant fluctuations and a higher RMSD value during the simulations (the RMSD averages of Cα atoms are shown in Table 2). This suggests that the DnaK^L484A^ variant may be more structurally unstable than DnaK^L484H^. In addition to differences in distance of arch structure residues, we also detected major differences in interaction energy, numbers of hydrogen bonds and hydrophobic interactions between the DnaK^L484A^ and DnaK^L484H^ variants (Table 2). In summary, DnaK^L484A^ showed substrate interaction characteristics similar to DnaK^WT^, while DnaK^L484H^ exhibited characteristics akin to DnaK^L484W^ (Table 2). These findings suggest that the structural and functional changes caused by L484W are likely due to the influence of the side chain rather than changes in hydrophobicity.

**Table 2:** Effects on substrate-binding interaction of different DnaK mutations.

Mutation	RMSD (nm)^a^	Interaction energy (kJ mol^−1^)^b^	Hydrogen bonds^c^	Hydrophobic interactions^c^	Distance of arch structure residues (nm)^d^	Number of hydrogen bonds between inner loops^d^
WT	0.246	−309.99	5.30	62.05	0.720	1.820
L484W	0.250	−435.06	6.86	72.48	0.715	1.667
L484A	0.282	−313.59	4.77	62.90	0.786	1.562
L484H	0.242	−498.96	7.40	79.86	0.697	0.319
G406S/L484W	0.263	−350.45	3.91	57.52	0.760	1.156
R445K/L484W	0.271	−364.29	5.75	63.90	0.730	1.354

a RMSD of Cα atoms in the stabilized MD structure.

b Interaction energy calculated during the SMD.

c Numbers of hydrogen bonds and hydrophobic interactions during the course of the SMD.

d Distance of arch structure residues and hydrogen bonds in between inner loops in stabilized MD structure.

### Increased SBD-substrate interactions are responsible for L484W effects

The results from the *in silico* structural analysis of DnaK^L484A^ and DnaK^L484H^ affords the opportunity to formally assess the functional impact of increased substrate binding upon DnaK. The interaction profiles would predict that DnaK^L484A^ would behave in a similar manner to DnaK^WT^ while DnaK^L484H^ would behave similarly to DnaK^L484W^ (Table 2).

We therefore exploited the yeast [*PSI* ^+^] system to test this hypothesis. Previous studies identified the Ssa1^L483W^ mutant (equivalent to DnaK^L484W^) as impairing propagation of the yeast prion [*PSI* ^+^] [12] and genetics studies suggest that this mutant binds more avidly to substrate [14]. To assess whether the SMD studies performed on DnaK could potentially be translated into meaningful comparisons with the yeast Ssa1 protein we constructed a molecular model of Ssa1 in complex with the same substrate as for DnaK, and performed the same SMD analysis on wild-type and L483 mutants as was carried out for DnaK (Text S1). Strikingly, the SMD analysis for Ssa1 is in excellent agreement with the SMD analysis for DnaK. Both Ssa1^WT^ and Ssa1^L483W^ display similar stability profiles (Figure S3) and Ssa1^L483W^ displayed an overall tighter affinity for substrate (Figure S4). Additionally Ssa1^L483W^ displayed an overall higher number of both hydrogen bonds and hydrophobic interactions over the time course of the SMD and the interaction profiles of the corresponding L483A and L483H mutants correlated well with their DnaK counterparts (Figures S5, Table S1).

**Figure 5:**
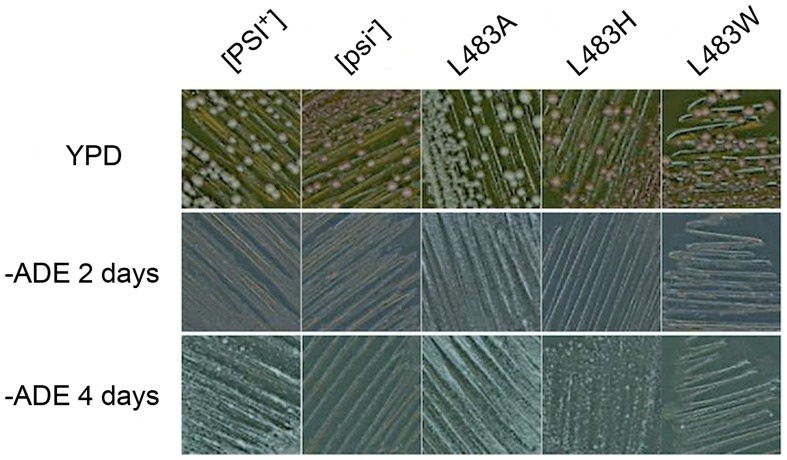
Assessment of [*PSI* ^+^] prion propagation in L483 mutants. Growth on adenine and color on YPD is indicative of yeast prion status. [*PSI* ^+^] and [*psi* ^−^] controls are in the presence of wild-type Ssa1. Strains are only expressing one type of cytosolic Hsp70, indicated by mutant type.

We therefore proceeded with *in vivo* studies in yeast and constructed Ssa1^L483A^ and Ssa1^L483H^ variants and tested their ability to propagate [*PSI* ^+^] when they were the sole cytosolic Hsp70 in the cell. Figure 5 demonstrates that similar to Ssa1^WT^ the Ssa1^L483A^ mutant can efficiently propagate [*PSI* ^+^] while similar to Ssa1^L483W^ the Ssa1^L483H^ variant has impaired [*PSI* ^+^] propagation; precisely what was predicted from the *in silico* DnaK studies. Additionally, we found that unlike Ssa1^L483W^ the Ssa1^L483H^ mutant could propagate [*PSI* ^+^], albeit at a much-reduced level compared to Ssa1^WT^ (Figure 5, -ADE 4 day panel), which may be a reflection of the wild-type level of hydrogen bonds between inner loop residues (Table S1) preventing the full impairment of the prion exhibited by L483W. The experimental confirmation of the *in silico* predictions demonstrates that MD and SMD simulations can be used to predict and design Hsp70 SBD mutants that may impair prion propagation *in vivo*.

Finally, previous experimental studies had identified second-site suppressor mutations of Ssa1^L483W^ that could restore [*PSI* ^+^] prion propagation [14]. Mutating conserved L483W second-site suppressors in DnaK provides the ideal opportunity for *in silico* testing for effects on SBD-substrate interactions when present in combination with DnaK^L484W^. We therefore assessed the structural consequences of introducing either of two mutations, G406S or R445K, in combination with L484W. MD and SMD simulations demonstrate that when either of these mutations is placed in combination with L484W there is a restoration of interaction energy and a reduction in the numbers of hydrogen bonds and hydrophobic interactions to levels similar to wild type (Table 2). This finding further implies that the major cause of functional and phenotypic changes in the Hsp70 L483/4W mutation is due to increased SBD-substrate interaction.

## Discussion

In this study we have taken an *in silico* approach to studying interactions between substrate and the SBD of the evolutionary conserved bacterial Hsp70 DnaK. Applying SMD simulations to DnaK^WT^ and the DnaK^L484W^ mutant has provided detailed structural information suggesting the L484W mutant has enhanced substrate-binding activity. Our structural results demonstrate that (*i*) the SBDs of DnaK^L484W^ and DnaK^WT^ have similar structural stability but the interaction energy of DnaK^L484W^ with substrate is stronger, (*ii*) the intermolecular hydrogen bonds and hydrophobic interactions between substrate and DnaK^L484W^ during the dissociation process is increased compared to DnaK^WT^, (*iii*) DnaK^L484W^ can more efficiently form the critical Ala429 to Met404 interaction that results in formation of a tighter arch to prevent substrate release compared with DnaK^WT^, (*iv*) the side chain structure, not hydrophobicity, play an important role in DnaK^L484W^-induced functional changes. The simulation results obtained in this study compare well to previous experimental studies addressing DnaK-substrate interactions [3] and also provide further evidence to suggest that the yeast Ssa1^L483W^ mutant and the equivalent DnaK mutant do indeed bind more avidly to substrate, as previously proposed [14].

Further validation of the use of SMD to study DnaK-substrate interactions comes from the analysis of the DnaK^L484A^ and DnaK^L484H^ mutants. Of particular note was the interaction profile of DnaK^L484H^, which clearly indicates that this mutant should have similar functional properties as the DnaK^L484W^ mutant. A key goal of this work was to provide insight into the functional changes in the yeast *SSA1-21* mutant that allow prion impairment. Through modeling wild-type Ssa1 and SBD mutants, and carrying out SMD simulations we clearly demonstrate that for the L483/4 residue of Ssa1/DnaK, the predicted functional changes that occur through mutation of this site are mimicked between the yeast and bacterial Hsp70 orthologs. Making the transition into the yeast system and demonstrating that when present in Ssa1 these two mutants significantly impair [*PSI* ^+^] prion propagation, is a major finding that suggests SMD can be used to design Hsp70 SBD mutants that would be predicted to impair prion propagation. Such an application may be important for the possible future design of small therapeutic molecules that may target the SBD of Hsp70. Given the importance of Hsp70 in the protein folding process and the array of human protein-folding diseases, SMD could aid significantly in the early stages of future drug design and development for these diseases.

Previous genetic studies have shown that the Ssa1-21 mutant causes a reduction in seed/propagon numbers *in vivo* [12], [13]. The most likely cause for prion impairment was proposed to be through an increased affinity for prion substrate exhibited by Ssa1-21, which would interfere with the prion replication cycle [13]–[15]. Strong genetic support exists for this proposal in that an array of second-site mutations have been isolated that can suppress the ability of Ssa1-21 to impair prion propagation and biochemical assessment of corresponding mutations in DnaK has previously shown that such mutations reduce substrate-binding affinity. Therefore it seems that mutations predicted to reduce substrate-binding efficiency suppress the prion impairing ability of Ssa1-21, thus suggesting that Ssa1-21 impairment of [*PSI* ^+^] is dependent on an increased substrate-binding activity of this mutant [14]. Additionally, deletion of co-chaperones known to promote substrate binding can also suppress the prion impairing ability of Ssa1-21 [15]. Further support for this hypothesis comes from our *in silico* studies on DnaK which not only predict increased substrate-binding affinity for the L484W variant but also predict a return to almost wild-type interaction profiles when L484W is in combination with either G406S or R445K. Both of these mutations were shown to suppress prion impairment exhibited by Ssa1^L483W^ [14].

A recent biochemical study found that compared to wild type the Ssa1-21 protein possesses hyperactive intrinsic ATPase activity and a reduced capacity to bind a peptide substrate [16]. This data is somewhat at odds with the *in vivo* activity of Ssa1-21 since the mutant protein is able to maintain cell growth similar to wild type and as yet no stress-related phenotypes have been associated with this mutant. Similarly, our *in silico* data is in excellent agreement with *in vivo* data and hypothesis of Ssa1-21 prion impairment [13]–[15] but somewhat at odds with the biochemical data regarding substrate-binding efficiency [16]. There are a number of complicating factors that could account for this apparent discrepancy. Firstly, the peptide substrates used in the two studies are different. Biochemical peptide-binding assays utilized the A7 peptide (Arg-Arg-Leu-Glu-Asp-Ala-Glu-Tyr-Ala-Ala-Arg-Gly) whereas our *in silico* study relied on the substrate used in the crystallization of DnaK SBD (Asn-Arg-Leu-Leu-Leu-Thr-Gly). These peptide substrates are very different in amino acid composition and in length and therefore complicate any direct comparison between the *in silico* and *in vitro* data. Secondly, the biochemical analysis was carried out using full-length Ssa1 protein in the ADP-bound state, while the *in silico* analysis used the isolated SBD of DnaK for which high-quality structural data is available. Hence, it is difficult to draw direct comparison between current biochemical data for Ssa1-21 and our computational data. Our data clearly predicts increased binding affinities for both DnaK^L484W^ and Ssa1^L483W^, which is supported by previous genetic studies and our own new *in vivo* data. Future biochemical analysis of these proteins should utilize a variety of peptides when assessing substrate-binding affinities and should also assess full-length and truncated versions of the chaperones, in order to understand the relationship between regulation of Ssa1 function *in vivo* and its biochemical properties *in vitro*.

In conclusion, we have demonstrated that SMD simulations can be used to study the process of substrate dissociation for DnaK and that in some cases this may apply to other Hsp70 orthologous. Of particular note is that SMD studies of Hsp70 may be used to predict and design new mutants that impair *in vivo* propagation of prions.

## Materials and Methods

### Model construction

In this study we used the solution structure of the substrate-binding domain from bacterial Hsp70 chaperone protein DnaK (393–507) in complex with the peptide NRLLLTG, obtained from the RCSD protein data bank (PDB code: 1Q5L) [21] as representing wild type protein. Appropriate models representing mutants of the L484 residue and subsequent second-site suppressor mutations, were constructed based upon the wild-type DnaK SBD structure using Swiss-Pdb Viewer [29] and subjected to Procheck analysis.

### MD and SMD simulations

Simulations were carried out using the GROMACS 4.0.7 software package [30] with constant number, pressure, temperature and periodic boundary conditions. The GROMOS96 43a1 [31] force field was applied in all simulations. The models were immersed in the cubic boxes filled with water molecules with a distance between peptides and box edges of at least 10 Å. Each cubic box was filled with approximately 10315 simple point-charge water molecules. The linear constraint solver (LINCS) method [32] was used to constrain bond lengths, allowing an integration step of 2 fs. Electrostatic interactions were calculated using the particle mesh Ewald algorithm with a distance cut off of 9 Å [33], [34]. Lennard-Jones potential was used for describing the short-range attractive and repulsive dispersion interactions with a 10 Å cut off. The temperature and pressure were coupled using V-rescale and Parrinello-Rahman algorithm respectively. The solvated and neutralized systems were then energy-minimized for 2,000 steps using the Steepest Descent algorithm to remove bad contacts. The backbone atoms of the structure were then fixed, while the side chains and solvent were allowed to move unrestrained for 80 ps. After equilibration several independent simulations were carried out over a 5 ns period at 300 K, pH7.0 and 1bar pressure. The coordinate trajectories were saved every 1 ps for subsequent data analysis.

Following 5 ns simulations models in the equilibrated state were used as input structures for the COM pulling method of GROMACS, to visualize pulling of the substrate through the substrate-binding domain cleft (Figure 1). In this process, a soft elastic spring of 300 kJ mol^−1^ nm^−2^ was associated with the substrate and applied in the direction along the cleft with a constant velocity. The substrate-binding domain was considered as the static reference in the pulling simulation. The trajectories were saved every 0.1 ps, and steering forces were recorded every 10 fs. Multiple SMD experiments were employed to each system for 2 ns.

### Analysis of trajectories

Analyses were performed using applications within the GROMACS package. All images were produced using the VMD [35]. The RMSD were calculated for all alpha-carbon atoms with reference to the first frame of the trajectories. Hydrogen bonds were calculated by a distance cutoff of 3.5 Å and an angle cutoff of 300°. Hydrophobic interactions were analyzed using Ligplot [36].

### Yeast strains, media and plasmids

The yeast strain used in this study was G400-1C (*MAT* **a** *ade2-1 SUQ5 kar1-1 his3 leu2 lys2 trp1 ura3 ssa1::KanMX*, *ssa2::HIS3*, *ssa3::TRP1*, *ssa4::URA3-1f*/pRDW10 [*PSI* ^+^]) [14]. The plasmid used was pC210, which is a *LEU2* based plasmid that contains the *SSA1* coding sequence under control of the constitutively active *SSA2* promoter [37]. Standard yeast media was used as previously described [38].

### Site directed mutagenesis and yeast genetics

Mutants of L483 (either W/H/A) were introduced into plasmid pC210 by site-directed mutagenesis (SDM) using the Quickchange kit (Stratagene) as recommended by the manufacturer. For L48W the primers L483W-F, GACTCTAACGGTATTTGGAATGTTTCCGCC and L483W-R GGCGGAAACATTCCAAATACCGTTAGAGTC were used, for L483H the primers L483H-F, GACTCTAACGGTATTCACAATGTTTCCGCC and L483H-R, GGCGGAAACATTGTGAATACCGTTAGAGTC, and for introducing L483A the primers L483A-F, GACTCTAACGGTATTGCGAATGTTTCCGCC and L483A-R, GGCGGAAACATTCGCAATACCGTTAGAGTC were used. Underlined codons indicate the SDM change. Ssa1 L483 mutants were assessed for [*PSI* ^+^] prion-impairing effects using the plasmid shuffle technique. Strain G400-1C was transformed with pC210 containing appropriate *SSA1* L483 mutation. Transformed cells were selected on medium lacking leucine. Transformants were re-streaked onto fresh medium and then replica plated onto medium containing limiting amounts of adenine and also 5-fluro-orotic acid (5-FOA), a chemical that selects against *URA* ^+^ cells and hence against the presence of the pRDW10 plasmid. Strains were then assessed for effects on the prion by using the well-defined red/white color assay based on the provision of adenine [39]. Briefly, the presence of [*PSI* ^+^] (the non-functional aggregated form of Sup35p) and *SUQ5* in strain G400-1c causes efficient translation read through of the *ochre* mutation in the *ade2-1* allele. Non-suppressed *ade2-1* mutants are Ade^−^ and are red when grown on medium containing limiting amounts of adenine due to the accumulation of a pigmented substrate of Ade2p. Partial suppression of *ade2-1* by [*PSI* ^+^] allows growth without adenine and eliminates the pigmentation [40]. The presence or absence of [*PSI* ^+^] was confirmed by mating experiments [39].

## Supporting Information

Figure S1 **The Cα RMSD of the DnaK^L484W^ (red) and DnaK^WT^ (black) as a function of simulation time.**
(JPG)

Figure S2 **Assessment of pulling velocity and spring constant in DnaK complex.** (A) Computed rupture forces as a function of pulling velocity *V*_pull_. The error bars give an estimated uncertainty. The dashed line shows a linear fit to the computed forces for *V*_pull_ less than 0.03 nm/ps. (B) Influence of different spring constants on the steering force of the DnaK complex.
(JPG)

Figure S3 **The Cα RMSD of the Ssa1^L483W^ (red) and Ssa1^WT^ (black) as a function of simulation time.**
(JPG)

Figure S4 **Time dependence of the interaction energy between peptide and Ssa1 cleft for Ssa1^L483W^ (red) and Ssa1^WT^ (black).**
(JPG)

Figure S5 **Changes in interaction forces as a function of simulation time.** (A), Temporal evolution of the hydrogen bonds number formed between the SBD of Ssa1 and peptide under the application of force for Ssa1^L483W^ (red) and Ssa1^WT^ (black) complexes. (B) Variation in the numbers of direct hydrophobic interactions of Ssa1^L483W^ (red) and Ssa1^WT^ (black) complexes in the SMD simulations. The calculation of interactions is recorded every 100 ps.
(JPG)

Table S1 **Effects on substrate-binding interaction during the SMD simulation for different Ssa1 and different L483 mutant derivatives.**
(PDF)

Text S1 **Construction of model for substrate-bound Ssa1.**
(PDF)
