# Supplementary figures and images for: Using Steered Molecular Dynamics to Predict and Assess Hsp70 Substrate-Binding Domain Mutants that Alter Prion Propagation
Source: PLoS Comput Biol. 2013 Jan 31;9(1):e1002896. doi: 10.1371/journal.pcbi.1002896 (PMC3561046; doi:10.1371/journal.pcbi.1002896)

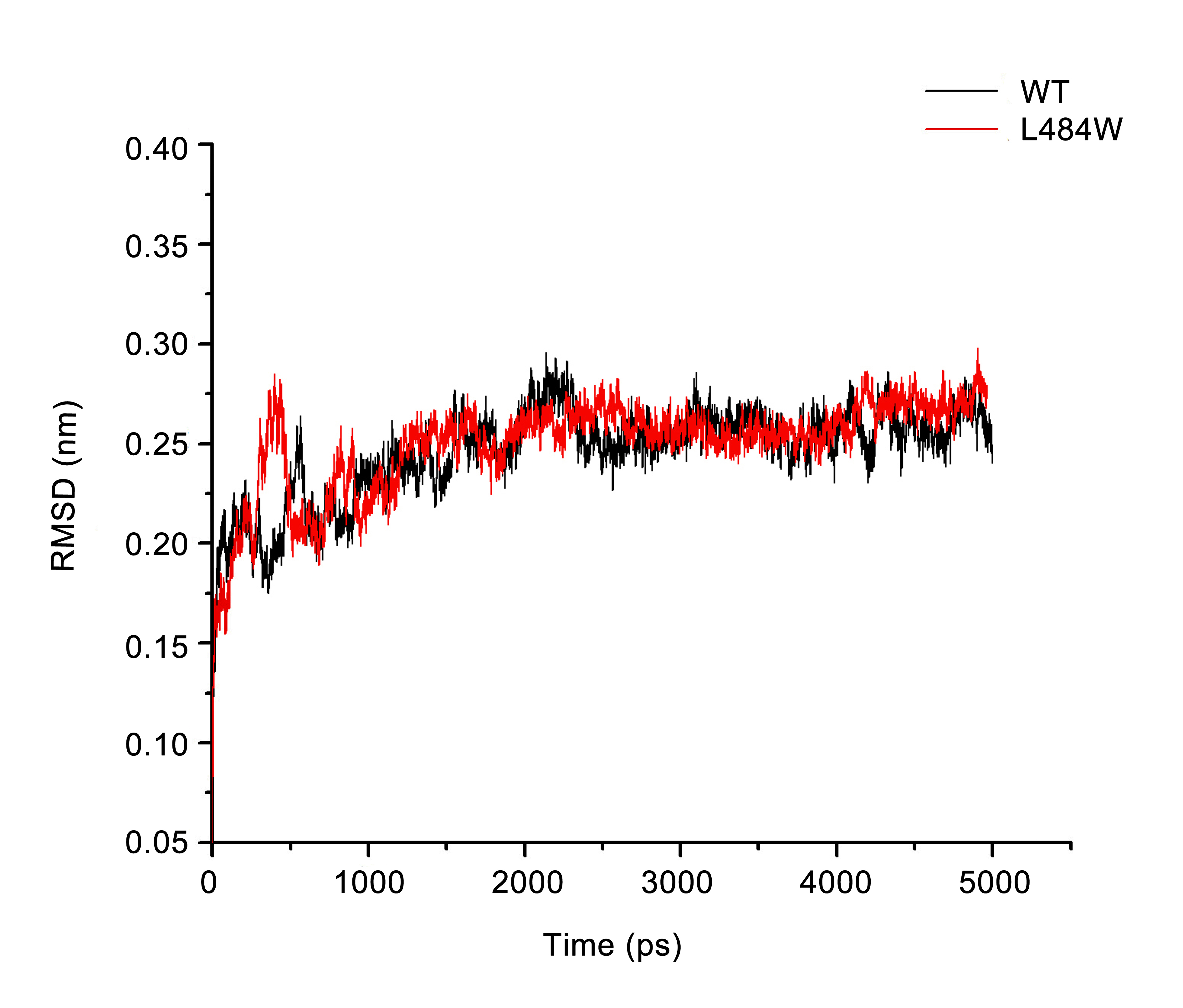

Supplement: Figure S1 — The Cα RMSD of the DnaKL484W (red) and DnaKWT (black) as a function of simulation time. (JPG) [file pcbi.1002896.s001.jpg]

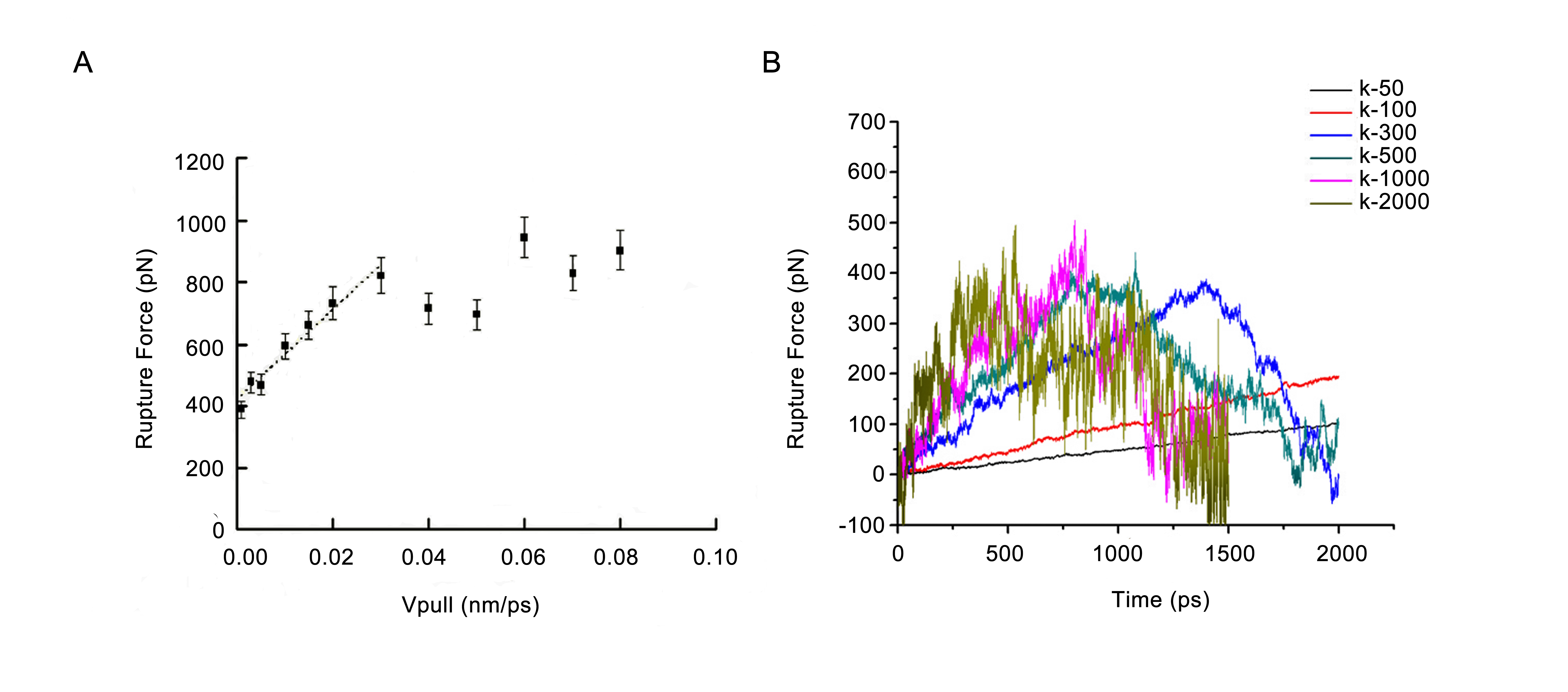

Supplement: Figure S2 — Assessment of pulling velocity and spring constant in DnaK complex. (A) Computed rupture forces as a function of pulling velocity V pull. The error bars give an estimated uncertainty. The dashed line shows a linear fit to the computed forces for V pull less than 0.03 nm/ps. (B) Influence of different spring constants on the steering force of the DnaK complex. (JPG) [file pcbi.1002896.s002.jpg]

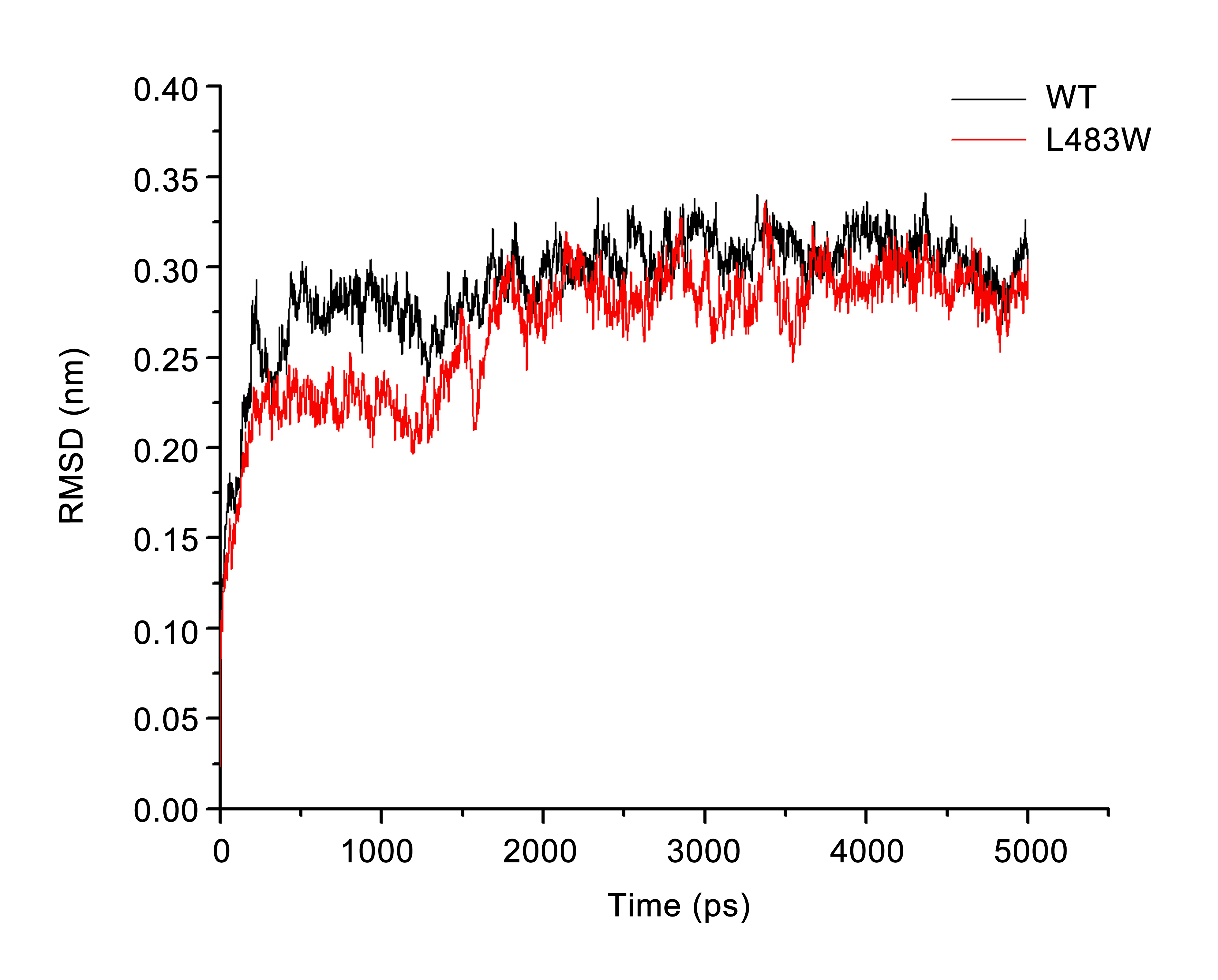

Supplement: Figure S3 — The Cα RMSD of the Ssa1L483W (red) and Ssa1WT (black) as a function of simulation time. (JPG) [file pcbi.1002896.s003.jpg]

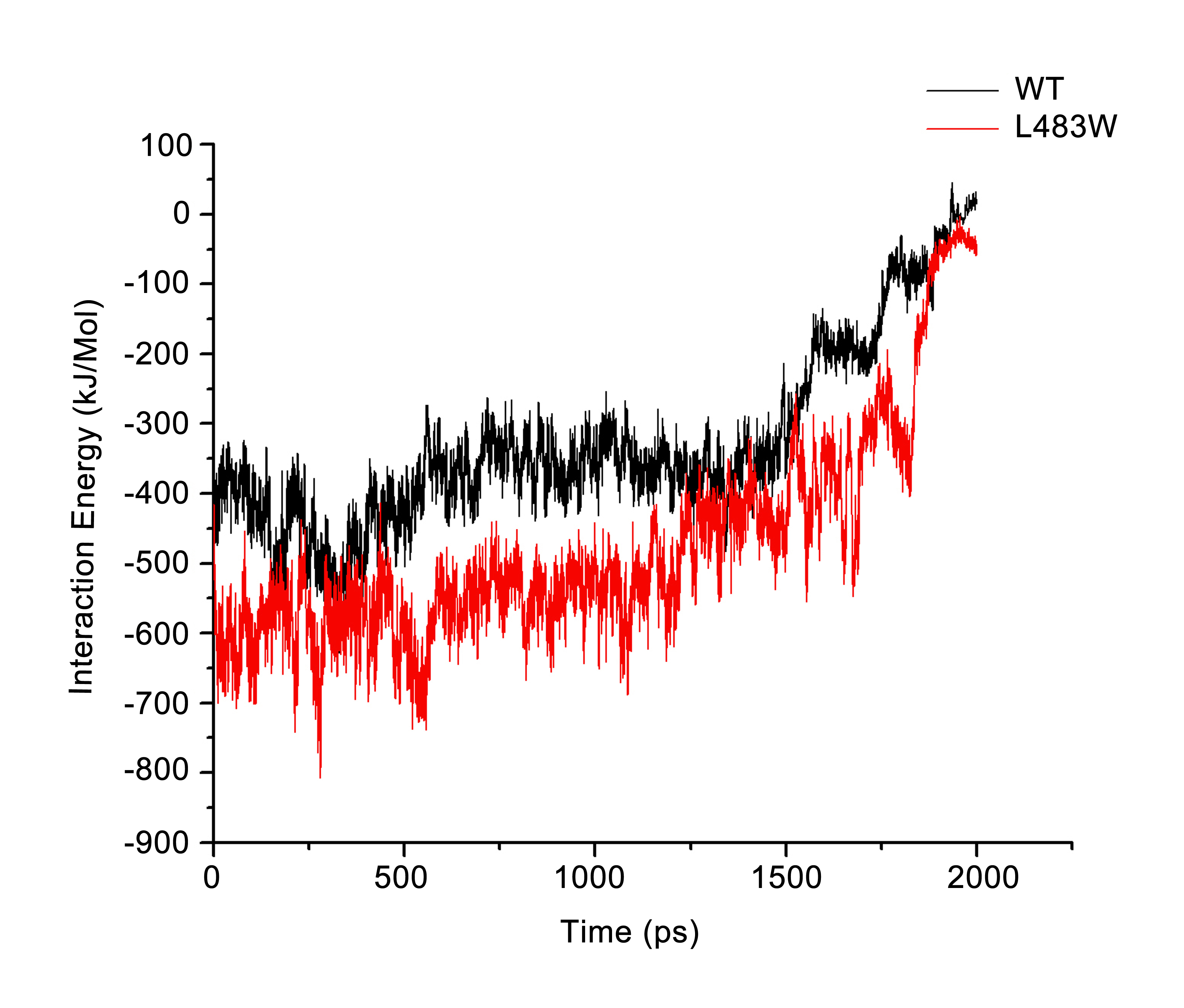

Supplement: Figure S4 — Time dependence of the interaction energy between peptide and Ssa1 cleft for Ssa1L483W (red) and Ssa1WT (black). (JPG) [file pcbi.1002896.s004.jpg]

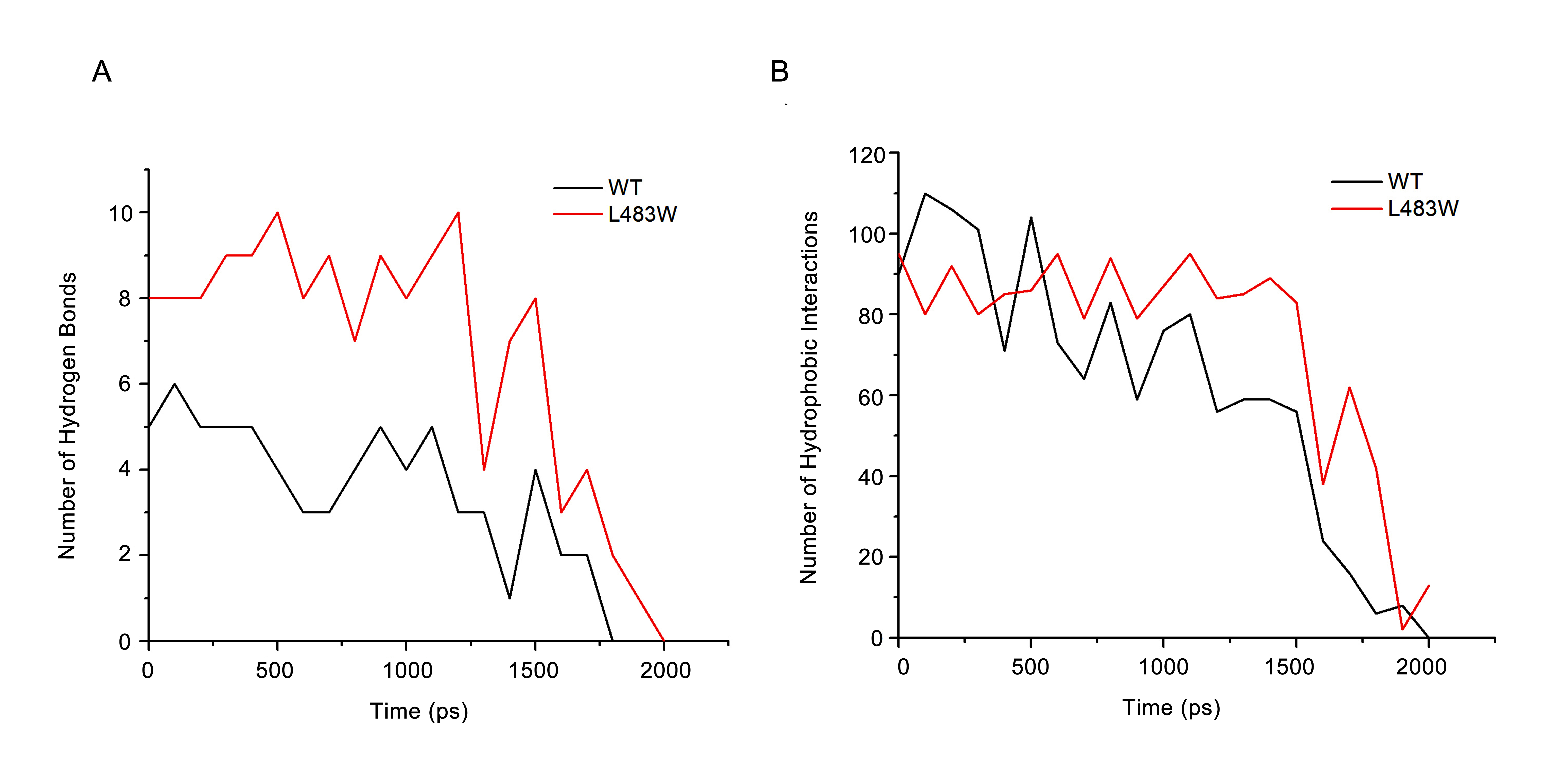

Supplement: Figure S5 — Changes in interaction forces as a function of simulation time. (A), Temporal evolution of the hydrogen bonds number formed between the SBD of Ssa1 and peptide under the application of force for Ssa1L483W (red) and Ssa1WT (black) complexes. (B) Variation in the numbers of direct hydrophobic interactions of Ssa1L483W (red) and Ssa1WT (black) complexes in the SMD simulations. The calculation of interactions is recorded every 100 ps. (JPG) [file pcbi.1002896.s005.jpg]
