# Supplementary material for: Using Steered Molecular Dynamics to Predict and Assess Hsp70 Substrate-Binding Domain Mutants that Alter Prion Propagation
Source: PLoS Comput Biol. 2013 Jan 31;9(1):e1002896. doi: 10.1371/journal.pcbi.1002896 (PMC3561046; doi:10.1371/journal.pcbi.1002896)
Supplement: Table S1 — Effects on substrate-binding interaction during the SMD simulation for different Ssa1 and different L483 mutant derivatives. (PDF) [file pcbi.1002896.s006.pdf]

Table S1 Effects on substrate-binding interaction of different Ssa1p mutations

Mutation	RMSD (nm)[a]	Interaction energy (kJ mol^{-1})[b]	Hydrogen bonds[c]	Hydrophobic interactions[c]	Distance of arch structure residues (nm)[d]	Number of hydrogen bonds between inner loops[d]
WT	0.291	-324.725	3.286	61.952	0.778	2.873
L483W	0.265	-472.013	6.714	73.571	0.582	2.444
L483A	0.265	-404.898	3.714	56.333	0.832	2.779
L483H	0.232	-518.677	7.048	87.047	0.664	2.944

[a]RMSD of Cα atoms in the stabilized MD structure.
[b]Interaction energy calculated during the SMD
[c]Numbers of hydrogen bonds and hydrophobic interactions during the course of the SMD.
[d]Distance of arch structure residues and hydrogen bonds in between inner loops in stabilized MD structure.
