# Supplementary material for: Using Steered Molecular Dynamics to Predict and Assess Hsp70 Substrate-Binding Domain Mutants that Alter Prion Propagation
Source: PLoS Comput Biol. 2013 Jan 31;9(1):e1002896. doi: 10.1371/journal.pcbi.1002896 (PMC3561046; doi:10.1371/journal.pcbi.1002896)
Supplement: Text S1 — Construction of model for substrate-bound Ssa1. (PDF) [file pcbi.1002896.s007.pdf]

Supporting Information

Model construction

In this study we used the substrate-binding domain from Ssa1p (392-507) in complex with the peptide NRLLLTG, which was constructed based upon the DnaK (PDB code: 1Q5L) (1), Hsc70 of *Bos taurus* (PDB code: 3C7N) (2) and Hsc70 of *Rattus norvegicus* (PDB code: 7HSC) (3) substrate-binding domain structures using Modeller 9v8 (4). Stereochemistry of the models was evaluated by Procheck. The best model was selected on the basis of DOPE score, GA341 score and Procheck. Appropriate models representing mutants of L483 residue mutations were constructed based upon the wild-type Ssa1p substrate-binding domain structure using Swiss-Pdb Viewer (5) and subjected to Procheck analysis.

References

1. Stevens SY, Cai S, Pellecchia M, Zuiderweg ERP (2003) The solution structure of the bacterial Hsp70 chaperone protein domain DnaK (393-507) in complex with the peptide NRLLLTG. Protein Science 12: 2588-2596.
2. Schuermann JP, Jiang J, Cuellar J, Llorca O, Wang L, Gimenez LE, Jin S, Taylor AB, Demeler B, Morano KA, Hart PJ, Valpuesta JM., Lafer EM and Sousa R. (2008) Structure of the Hsp110:Hsc70 Nucleotide Exchange Machine. Mol Cell 31(2): 232-243.
3. Morshauser RC, Hu W, Wang H, Pang Y, Flynn GC, Zuiderweg ERP. (1999) High-resolution solution structure of the 18 kDa substrate-binding domain of the mammalian chaperone protein Hsc70. J Mol Bio 289: 1387-1403.
4. Sali A and Blundell TL (1993) Comparative protein modelling by satisfaction of spatial restraints. J Mol Biol 234: 779-815.
5. Guex N, Peitsch MC (1997) SWISS-MODEL and the Swiss-Pdb Viewer: an environment for comparative protein modeling. Electrophoresis 18: 2714-2723.
